# lncRNA BDNF-AS Attenuates Propofol-Induced Apoptosis in HT22 Cells by Modulating the BDNF/TrkB Pathway

**DOI:** 10.1007/s12035-022-02757-y

**Published:** 2022-03-26

**Authors:** Yu-Hai Xu, Yuan Luo, Jiang-Bei Cao, Yan-Hong Liu, Yu-Xiang Song, Xiao-Ying Zhang, Qiang Fu, Wei-Dong Mi, Hao Li

**Affiliations:** 1 Present Address: 1st Medical Center of Chinese PLA General Hospital, 28th Fuxing Road, Haidian District, Beijing, 100853 China; 2 Air Force Medical Center, PLA, 30th Fucheng Road, Haidian District, Beijing, 100142 China

**Keywords:** Propofol, lncRNA, BDNF-AS, Hippocampus, Apoptosis

## Abstract

Propofol is widely used as an intravenous anesthetic in clinical practice. Previous studies have indicated that propofol induces apoptosis in neurons. Brain-derived neurotrophic factor (BDNF), a neurotrophic factor, is associated with neuronal apoptosis. BDNF-AS, a relatively conserved long non-coding RNA, can reverse the transcription of BDNF. This study aimed to investigate the involvement of BDNF-AS in propofol-induced apoptosis in HT22 cells. HT22 cells were treated with various concentrations of propofol at different time points. BDNF-AS was silenced using BDNF-AS-targeting siRNA. TrkB was antagonized by the TrkB inhibitor, ANA-12. Flow cytometry, quantitative reverse-transcription PCR, and western blotting were performed to analyze apoptosis and the expression of genes and proteins, respectively. In propofol-treated HT22 cells, BDNF-AS was upregulated, and BDNF was downregulated in a time- and dose-dependent manner. BDNF-AS downregulation mediated by siRNA mitigated apoptosis, upregulated the expression of Bcl-2, and downregulated the expression of Bax and caspase-3, 7, and 9. ANA-12 downregulated the expression of Bcl-2, upregulated the expression of Bax and caspase-3, 7, and 9, and increased apoptosis. Our study implied that inhibition of BDNF-AS can decrease propofol-induced apoptosis by activating the BDNF/TrkB pathway. Thus, the BDNF-AS-BDNF/TrkB signaling pathway may be a valuable target for treating propofol-induced neurotoxicity.

Propofol is a type of intravenous anesthetic drug widely used in clinical practice. In recent years, it has been shown that learning disabilities induced by long-term high-dose infusion of propofol are associated with neuronal apoptosis [1–3]. Several mechanisms have been identified to demonstrate propofol-induced apoptosis, including activation of pro-apoptotic factors, inhibition of apoptosis suppressor factors, and mitochondrial dysfunction [4–6]. Although various mechanisms are thought to be involved, the exact mechanism of propofol-mediated neuronal damage in the hippocampus remains to be determined.

Brain-derived neurotrophic factor (BDNF) is a neurotrophic factor that is a key regulator of neurite and glial maturation and synaptic differentiation in the central nervous system [7]. It regulates the growth and differentiation and maintains the vitality and function of neurons [8]. Some studies show that the neurotoxicity of propofol is related to its effect on the expression of BDNF. Karen et al. [9] found that acute neurotrophic disorder and neuronal apoptosis in neonatal rat brains induced by propofol exposure are associated with the downregulation of BDNF in the cortex. Popic et al. [10] reported that BDNF and downstream kinase Akt/ERK signaling is an essential component of the endogenous defense mechanism used by the developing brain to protect itself from potential propofol-induced damage.

Long non-coding RNAs (lncRNAs) are a group of non-coding RNAs of more than 200 nucleotides, playing important roles in regulating epigenetic, transcriptional, or post-transcriptional gene expression and many other cellular functions [11]. lncRNAs are involved in many aspects of neurodevelopment or neurological diseases, such as neurogenesis and neurodegeneration [12–14]. Modarresi et al. [15] found that downregulation of BDNF-AS can upregulate endogenous BDNF, promote the growth of neurites, and induce neuronal differentiation. Bohnsack et al. [16] reported that in patients with alcohol use disorders, increasing BDNF-AS reduces the expression of related BDNF, thereby affecting brain development and synaptic plasticity through the recruitment of EZH2 and the position of reactive H3K27me3 marks.

In this study, we explored the biological significance of BDNF-AS in propofol-stimulated HT22 cells and the possible signaling pathways involved in propofol-induced HT22 cell apoptosis.

## Materials and Methods

### Cell Culture and Reagents

HT22 mouse hippocampal neuronal cells were obtained from BeNa Culture Collection (Beijing, China) and cultured in Dulbecco’s modified Eagle’s medium (Sigma–Aldrich, Shanghai, China) supplemented with 10% fetal bovine serum (EVERYGREEN, Hangzhou, China) and 1% penicillin/streptomycin (Sigma). The cells were incubated in a humidified incubator containing 5% CO_2_ at 37 °C and sub-cultured on reaching 90% confluence. Propofol (Sigma–Aldrich, Shanghai, China) and TrkB inhibitor ANA-12 (Selleckchem, Shanghai, China) were dissolved in dimethyl sulfoxide (DMSO; Sigma–Aldrich, Shanghai, China), and the concentration of DMSO was adjusted to 0.01%. In total, 5 μM ANA-12 was added to the HT22 cell culture to inhibit TrkB.

### Study Design

HT22 cells were treated with different concentrations (12.5 μM, 25 μM,50 μM, 100 nμM) of propofol for 24 h, and other groups of cells were treated with 50 μM propofol for different times (12 h, 24 h, 48 h). In the following experiments, the optimal treatment time and concentration of propofol were used to investigate potential mechanisms. To investigate the potential mechanism of propofol-induced apoptosis in HT22 cells.

### Cell Transfection

Small interfering RNA against BDNF-AS (si-RNA) and si-RNA negative control (NC) were synthesized and purchased from Ribobio (Guangzhou, China), according to a previous study [17]. According to the manufacturer’s protocol, 200 nM si-RNAs and non-specific control siRNA were transfected into HT22 cells using Lipofectamine 2000 (Invitrogen, Carlsbad, USA) for 24 h. The efficiency of gene interference was validated by quantitative reverse-transcription PCR (qRT-PCR).

### Flow Cytometry

Cell apoptosis was evaluated using the FITC/PI Apoptosis Kit (Solarbio, Beijing, China). According to the manufacturer’s protocol, the HT22 cells were collected, washed twice with cold phosphate buffered saline, and re-suspended in 1 × binding to reach a density of 1 × 10^6^ cells/mL. The suspension cells were mixed with Annexin-V and PI without any light for 15 min and then applied to a flow cytometer (Beckman Coulter, Fullerton, CA, USA).

### qRT-PCR

Total RNA was extracted using Trizol reagent (Invitrogen, Carlsbad, USA), and the concentration and purity of RNA were detected with the help of a UV spectrophotometer (Thermo Fisher Scientific, USA). cDNA was synthesized using a cDNA Transcription Kit (Takara, Tokyo, Japan). qRT-PCR was performed using the SYBR Green PCR Master Mix kit (Takara). The primer sequences of BDNF were as follows: forward, 5′-GGGA CCGGT TTG TGT-3′; reverse, 5′-TTG CTT TTT CAT GGG GGC A-3′. The primer sequences of BDNF–AS were forward, 5′-TACCACAAGGTACCAACCATATATG-3′, reverse: 5′-CATGTGGTTCTGTTTCAATGCCC-3′. GAPDH was the internal control, and the relative expression of BDNF-AS and BDNF was calculated by the 2^−ΔΔCt^ method.

### Western Blot Assay

Total proteins were separated from cells using RIPA Lysis Buffer (Beyotime, Shanghai, China), and protein concentrations were detected using the BCA Kit (Beyotime, Shanghai, China). After quantity measurement, the proteins were separated by 12% SDS-PAGE (Invitrogen, Carlsbad, USA) and transferred to a polyvinylidene difluoride membrane. The membranes were blocked for 60 min at room temperature and incubated with the following antibodies overnight at 4 °C: antibodies against BDNF (1:500), TrkB (1:500), phospho-TrkB (*p*-TrkB, o 1:500), caspase-3,7,9 (1:1000), GAPDH (1:1000), Bax (1:2000), and bcl-2 (1:1000) were obtained from Cell Signaling Technology (Danvers, USA). The next day, the membrane was washed with TBST and incubated with secondary antibodies. Protein signals were detected using an enhanced chemiluminescence system (Beyotime, USA).

### Statistical Analysis

All experiments were independently repeated at least three times. All data are expressed as mean ± standard deviation (SD). SPSS software (version 20.0, SPSS, USA) was used for the statistical analysis. One-way analysis of variance was used to compare the differences between the multiple groups. The differences between the two groups were compared using the Student’s *t*-test with Bonferroni correction. Statistical significance was set at *P* < 0.05.

## Results

### Propofol Induced Apoptosis and Inversely Regulated the Expression of BDNF-AS and BDNF in HT22 Cells in a Dose- and Time-Dependent Manner

**Fig. 1:**
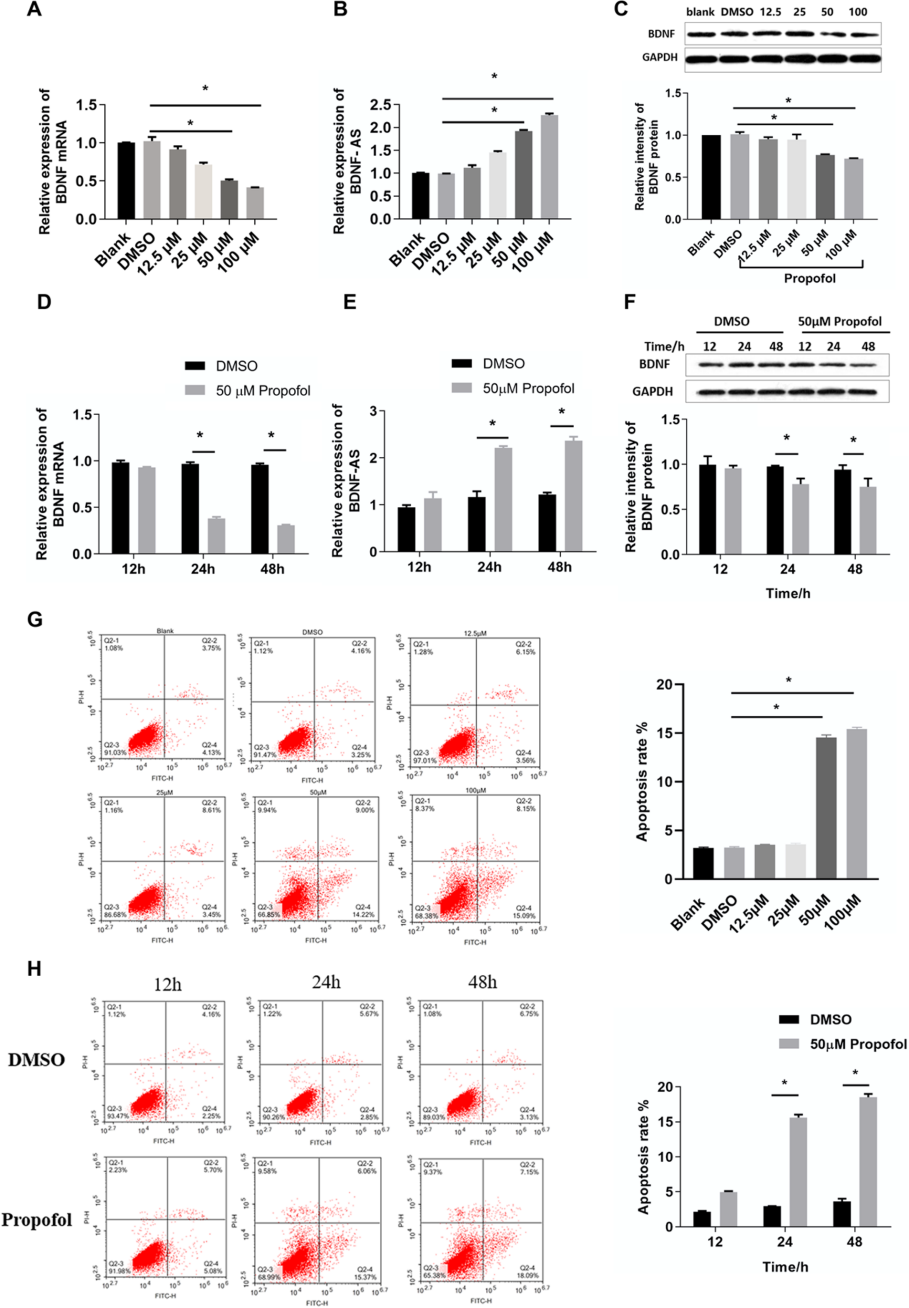
HT22 cells were treated with propofol at a concentration of 0, 12.5, 25, 50, and 100 μM for 24 h; qRT PCR was performed to evaluate the gene expression of BDNF (**A**) and BDNF-AS (**B**). Western blotting was performed to evaluate the expression of BDNF (**C**). HT22 cells were treated with propofol at a concentration of 50 μM for 12, 24, and 48 h; qRT PCR was performed to evaluate the endogenous gene expression level of BDNF (**D**) and its natural antisense long non-coding RNA BDNF-AS (**E**). Western blotting was performed to evaluate the expression of BDNF (**F**). Flow cytometry was used to appraise the apoptosis of the HT22 cells (**G**–**H**) (*n* = 4, *P < 0.05, compared with the DMSO group)

qRT-PCR showed that propofol (12.5–100 μm) significantly reduced the expression of BDNF in the cells (Fig. 1A, *P < 0.05), while significantly increasing the expression of BDNF-AS (Fig. 1B, *P < 0.05). Western blotting analysis showed that the propofol treatment suppressed BDNF protein expression in a dose-dependent manner (Fig. 1C, *P < 0.05). HT22 cells were treated with 50 μM propofol for different periods (12–48 h). We found that as time increased, endogenous BDNF expression decreased (Fig. 1D, *P < 0.05), and BDNF-AS expression increased (Fig. 1E, *P < 0.05). Western blotting analysis showed that the propofol treatment suppressed BDNF protein expression in a time-dependent manner (Fig. 1F, *P < 0.05). Flow cytometry showed that when the concentration of propofol reached 50 μm, the apoptosis rate began to show statistical difference compared with DSMO group and when 50 μm propofol stimulated HT22 cells for more than 24 h, the apoptosis rate began to show statistical difference (Fig. 1G,H, *P < 0.05). Therefore, our study shows that BDNF and BDNF-AS are inversely regulated in a dose- and time-dependent manner by propofol in mouse hippocampal neuronal cell lines.

### Downregulation of BDNF-AS Protected HT22 Cells from Propofol-Induced Apoptosis by Upregulating BDNF

**Fig. 2:**
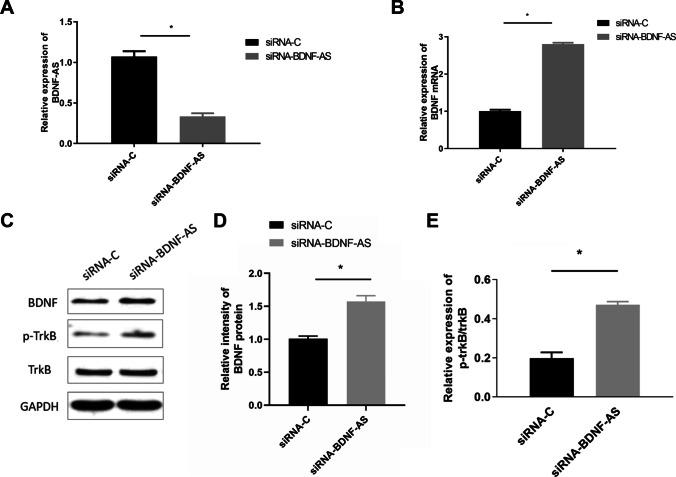
After being transfected with siRNA-C or siRNA-BDNF-AS, qRT-PCR was performed to detect BDNF-AS and BDNF mRNA expression from HT22 cells (**A**, **B**). Western blotting was performed to detect the expression of BDNF and *p*-TrkB/TrkB proteins (**C**) (*n* = 4, *P < 0.05)

To investigate the role of BDNF-AS in HT22 cell propofol-induced apoptosis, HT22 cells were transfected with BDNF-AS-specific siRNA (siRNA-BDNF-AS) or non-specific control siRNA (siRNA-C) for 24 h. qRT-PCR revealed that BDNF-AS-specific siRNA successfully suppressed the expression of BDNF-AS (Fig. 2A, *P < 0.05) but significantly upregulated the expression of the BDNF gene (Fig. 2B, *P < 0.05). HT22 cells were treated with 50 μM propofol for 24 h. Western blotting revealed that BDNF was upregulated, and phosphorylation of TrkB was significantly increased in cells transfected with BDNF-AS-specific siRNA (Fig. 2C, *P < 0.05).

### Propofol Induces HT22 Cell Apoptosis Through BDNF/TrkB Signaling Pathway

**Fig. 3:**
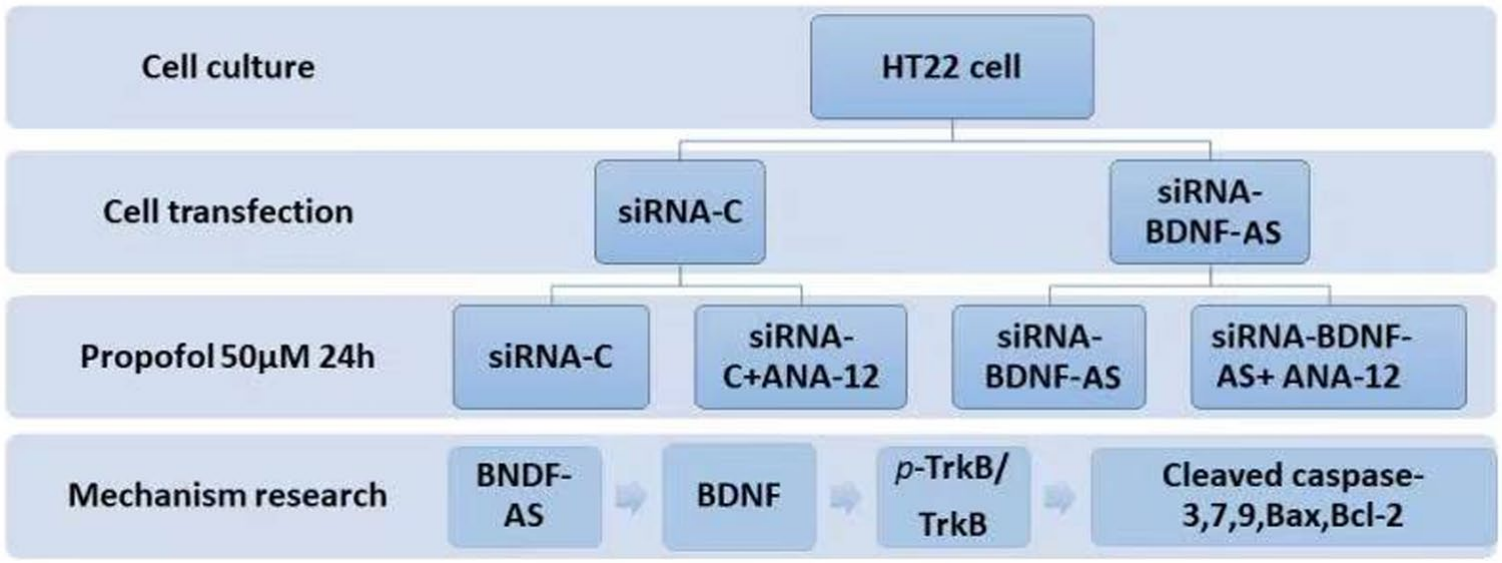
A TrkB inhibitor (ANA-12) was used to investigate if HT22 cell apoptosis was mediated by the BDNF/TrkB signaling pathway

**Fig. 4:**
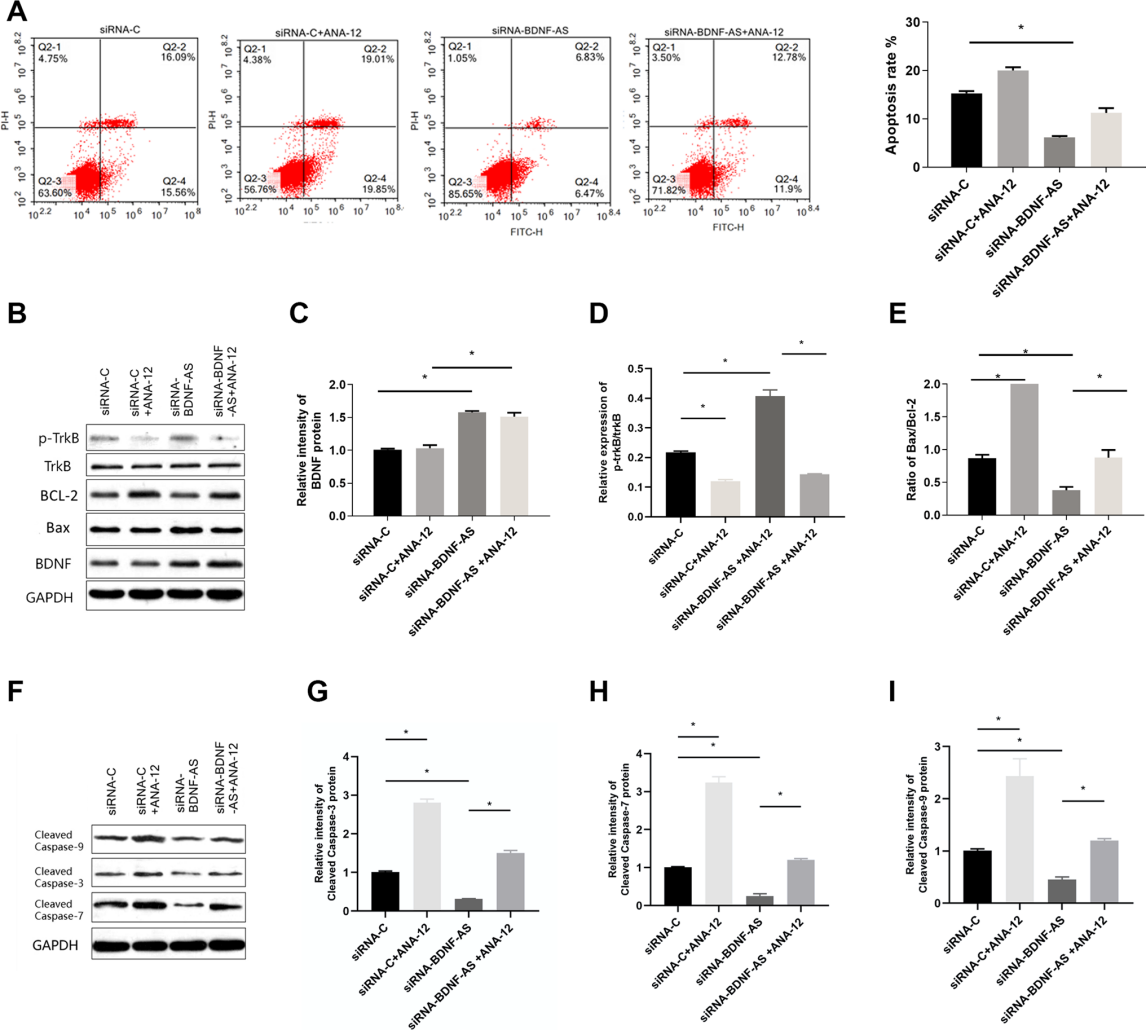
Following transfection with siRNA-BDNF-AS or siRNA-C for 24 h, ANA-12 was used in the cells in the presence of 50 μM propofol for 24 h. Flow cytometry was used to appraise the apoptosis of the HT22 cells (**A**). The protein levels of BDNF, TrkB, *p*-TrkB, Cleaved caspase-3, 7, and 9, Bax, and Bcl-2 were detected by western blotting (**B**–**I**) (*n* = 4, *P < 0.05)

To determine whether propofol-induced HT22 cell apoptosis was mediated by the BDNF/TrkB signaling pathway, a TrkB inhibitor (ANA-12) was used to block the BDNF/TrkB pathway, following transfection with BDNF-AS-specific siRNA or non-specific control siRNA for 24 h. ANA-12 (1 μM) was added to the cells in the presence of 50 μM propofol for 24 h (Fig. 3). Flow cytometry showed that apoptosis of HT22 cells was inhibited by BDNF-AS suppression, and ANA-12 increased the apoptosis rate (Fig. 4A, *P < 0.05). The inhibition of BDNF-AS increased the expression of BDNF protein (Fig. 4C, *P < 0.05), and significantly increased TrkB phosphorylation, while TrkB phosphorylation was inhibited by ANA-12 (Fig. 4D, *P < 0.05). The inhibition of BDNF-AS decreased the expression of pro-apoptotic markers (caspase-3, 7, 9 and Bax) and increased the expression of the anti-apoptotic marker Bcl-2, and the administration of ANA-12 mitigated the effect of BDNF, increased cleaved caspase-3, 7, and 9 and Bax expression and decreased Bcl-2 expression (Fig. 4B–I, *P < 0.05). The expression profiles of caspase-3,7, and 9 and Bax/Bcl-2 ratio were consistent with the apoptosis results (Fig. 4A, F–I, *P < 0.05). These results indicate that BDNF-AS negatively regulates BDNF expression through the BDNF/TrkB signaling pathway, thus affecting the apoptosis of propofol-induced hippocampal neurons.

## Discussion

Propofol is widely used as an intravenous anesthetic in patients under clinical anesthesia. Although the safety of propofol is controversial, it is still widely used in obstetrics, pediatric surgery, and even neonatal anesthesia [18]. In recent years, some studies have shown that propofol may cause nerve cell injury and neuronal apoptosis. For example, in in vivo and in vitro models, Pearn et al. found that propofol induced neurotoxicity by the loss of RhoA-dependent dendritic spines and impaired retrograde axonal transport of BDNF [19]. An in vivo study by Cao et al. demonstrated that propofol changes the differentiation of neural stem cells (NSCs) through the miR-124/SP1/CDKN1B axis, resulting in neuronal damage [20]. Consistent with the above findings, we found that propofol was cytotoxic and caused apoptosis in HT22 cells. When the concentration of propofol was higher than 50 μM and the stimulation time was more than 24 h, there was a significant difference in the apoptosis rate of HT22 cells, suggesting that long-term and high-dose infusion of propofol could be harmful to hippocampal cells.

Studies have shown that BDNF, which is found at relatively high levels in the hippocampus, is a key regulatory factor in neuronal cell survival and is also involved in nerve cell apoptosis. Matusica et al. [21] indicated that BDNF enables P75 (NTR)-derived trophic cell permeapeptides (c29) to promote neuronal survival through the TrkB-dependent signaling pathway in vitro and in vivo. The BDNF/TrkB pathway in learning and memory [22], chronic pain [23], and retinopathy [24] has been deeply studied, but there are few studies on neuronal apoptosis. BDNF-AS is a natural antisense transcript that inhibits the transcription of BDNF [15, 25]. Zhong et al. [26] reported that knockdown of BDNF-AS upregulated the expression of BDNF and attenuated nerve cell apoptosis. Zhang et al. [27] reported that knockdown of lncRNA BDNF-AS inhibited neuronal cell apoptosis in acute spinal cord injury. In line with the results of a previous study, we discovered that the expression of BDNF mRNA and protein was downregulated by propofol, and BDNF-AS was upregulated by propofol in HT22 cells, and siRNA-mediated BDNF-AS downregulation could significantly upregulate endogenous BDNF expression and enhance TrkB protein phosphorylation.

In this study, we found that propofol increased the abundance of BDNF-AS and decreased the expression of BDNF in HT22 cells in a dose- and time-dependent manner and when the concentration of propofol was more than 50 μM and the stimulation time was more than 24 h, the apoptosis rate increased significantly suggesting that BDNF-AS might participate in propofol-induced neurotoxicity. We conducted BDNF-AS knockout experiments to verify whether propofol damaged nerve cells via BDNF-AS and found that the promoting effects of propofol processing on apoptosis were reversed by BDNF-AS knockout, suggesting that BDNF-AS is involved in propofol-induced neurotoxicity. Additionally, we found that knockout of BDNF-AS increased the phosphorylation of TrkB and apoptosis of HT22 cells, leading to the hypothesis that neurotoxicity induced by propofol in HT22 cells is mediated by BDNF/TrkB axis. To verify this hypothesis, we used an antagonist of the TrkB receptor, ANA-12, and found that ANA-12 can increase propofol-related apoptosis. The results of Bax/Bcl-2 ratio and cleaved caspase-3, 7, and 9 also confirmed the hypothesis.

There are some limitations to our study. First, all the results were based on in vitro cell line experiments, with no in vivo validation. In vivo, many different types of nerve cells interact and influence each other. Our study consisted of a cell line culture, which may have prevented us from investigating the actual effects of propofol on neurons. Second, we used cell death as the final endpoint; however, propofol may adversely affect surviving neurons that in turn affect cell function, such as disrupt the differentiation of NSCs [20] and impair hippocampal synaptic plasticity [28].

## Conclusions

Overall, we demonstrated that propofol induces cell apoptosis in HT22 mouse hippocampal neuronal cells, which can be alleviated by downregulation of lncRNA BDNF-AS, while BDNF protected HT22 cells from propofol, blocking the BDNF-TrkB pathway and aggravating propofol-induced apoptosis. This study suggests that long-term and high-dose infusion of propofol could be harmful to hippocampal cells and the BDNF-AS/BDNF/TrkB pathway may be a potential target to protect hippocampal neuronal cells against propofol. Our future research will focus on TrkB downstream signaling pathways in propofol-induced nerve injury.

## Data Availability

All data in the current study are available from the corresponding authors on reasonable request.
